# Spikebot: A Multigait Tensegrity Robot with Linearly Extending Struts

**DOI:** 10.1089/soro.2023.0030

**Published:** 2024-04-08

**Authors:** Jinwook Jeong, Injoong Kim, Yunyeong Choi, Seonghyeon Lim, Seungkyu Kim, Hyeongwoo Kang, Dylan Shah, Robert Baines, Joran W. Booth, Rebecca Kramer-Bottiglio, Sang Yup Kim

**Affiliations:** ^1^ Department of Mechanical Engineering, Sogang University, Seoul, Republic of Korea.; ^2^ Department of Mechanical Engineering and Materials Science, Yale University, New Haven, Connecticut, USA.

**Keywords:** tensegrity, soft robotics, puffer fish, pneumatic actuation, multigait

## Abstract

Numerous recent research efforts have leveraged networks of rigid struts and flexible cables, called tensegrity structures, to create highly resilient and packable mobile robots. However, the locomotion of existing tensegrity robots is limited in terms of both speed and number of distinct locomotion modes, restricting the environments that a robot is capable of exploring. In this study, we present a tensegrity robot inspired by the volumetric expansion of Tetraodontidae. The robot, referred to herein as Spikebot, employs pneumatically actuated rigid struts to expand its global structure and produce diverse gaits. Spikebot is composed of linear actuators that dually serve as rigid struts linked by elastic cables for stability. The linearly actuating struts can selectively protrude to initiate thrust- and instability-driven locomotion primitives. Such motion primitives allow Spikebot to reliably locomote, achieving rolling, lifting, and jumping. To highlight Spikebot's potential for robotic exploration, we demonstrate how it achieves multi-dimensional locomotion over varied terrestrial conditions.

## Introduction

By combining the benefits of rigid and soft components, biological systems dynamically change their stiffness and gait, allowing them to easily navigate complex environments. Taking cues from systems as varied as the human musculoskeletal system to the carapace-plastron structure of turtles,^1–4^ engineers have begun creating hybrid systems that combine the benefits of soft and rigid systems to create efficient and lightweight robots.^5–9^

One particularly interesting class of structures, namely tensegrities that consist of rigid struts and compliant cables, has been shown to be the optimally lightweight structure under compressive loads,^10^ leading researchers to develop a new field of tensegrity robotics.^11–13^

Most tensegrity robots to date have adopted a six-strut topology with a roughly spherical shape, known as Jessen's icosahedron, and exhibit a controlled rolling locomotion by shifting the center of gravity^14–16^ outside of the polygon of stability. The center of gravity of icosahedron tensegrity robots has previously been achieved by modifying tensile forces of cables,^17–19^ adjusting the length of the struts,^20,21^ or inflating membranes that constitute faces of the polyhedron.^22,23^

For example, earlier versions of tensegrity robots utilize motors attached to the end of their struts to control cable tensions,^18,19^ allowing the robot to be geometrically unstable and roll. Subsequent studies have expanded the motion primitives of tensegrity robots to vibration,^24,25^ flying,^26,27^ and shape memory.^28–30^

However, such motion primitives have limited the speed and efficiency of tensegrity robots, because the deformation of the overall shape often consumes more energy than what is needed for locomotion. In addition, the deformation of the internal cavity reduces the robots' payload-carrying capabilities.

To achieve higher locomotion speeds, several studies reported jumping locomotion using additional bulky actuators,^26,31^ allowing potential gains in overall mission energy efficiency and speed. Yet, as demonstrated to date, jumping tensegrity motions tend to be unstable and suffer from low repeatability.

A key feature of tensegrity robots is their rigid struts. Since the struts provide ample space to implement conventional actuation mechanisms, a tensegrity may be equipped with linear actuators to unlock a novel locomotion mode. Up to now, the actuation of struts has only been exploited to induce structural deformation of tensegrity robots. Consequently, tensegrity robots have been unable to fully utilize actuation originating from their struts^15^ and seamlessly implement other types of conventional actuators.

To overcome these limitations and create a tensegrity capable of multiple repeatable locomotion modes, we present a new motion primitive for tensegrity robots that is decoupled from structural deformation. Inspired by the Tetraodontidae, which takes in external fluid to realize the rapid volumetric expansion of a globe,^32^ we propose a tensegrity robot (furthermore referred to as Spikebot) that can extend its rods out of its nominally convex hull while retaining its original internal shape. Node-originated extension of rods creates a similar effect to volumetric expansion of the tensegrity, serving as an effective and efficient locomotion primitive.

The new locomotion primitives attained by Spikebot solve notable challenges of deformation-based tensegrity robots, by enabling rapid rolling and jumping behaviors. Spikebot is capable of selectively actuating each strut at high velocity to elicit several locomotion gaits. We characterize the stability and controllability of each locomotion gait, and showcase the utility of Spikebot as a mobile robot over several terrestrial conditions, including inclines and obstacles.

## Design and Manufacture

### Tetraodontidae-inspired design of tensegrity

**FIG. 1.**
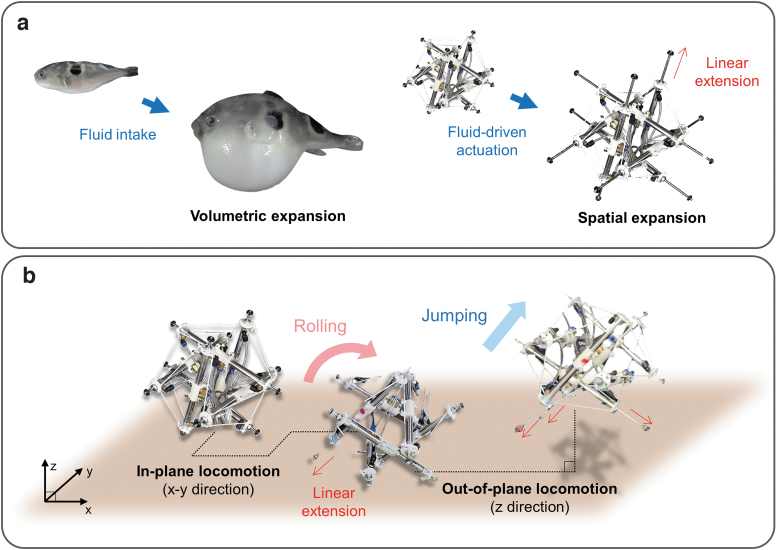
Overview of a multigait tensegrity robot referred to herein as Spikebot, inspired by the volumetric expansion of Tetraodontidae. **(a)** The working principle behind the volumetric expansion of Tetraodontidae and Spikebot, respectively. Spikebot utilizes the linear extension of each strut to realize spatial expansion similar to that of Tetraodontidae. **(b)** Schematic of the multi-mode locomotion of Spikebot capable of in-plane (*x*–*y* direction) rolling and out-of-plane (*z*-direction) jumping motions.

Spikebot mimics the fluid-driven volumetric expansion^33–35^ of a Tetraodontidae, as shown in Figure 1. The Tetraodontidae intakes fluid to rapidly and efficiently morph its body shape. Likewise, Spikebot increases its bounding volume by extending its pneumatically-actuated struts (Fig. 1a). By utilizing independently controlled linear actuators, we locally distribute changes in bounding volume to impart diverse locomotion modes, such as rolling and jumping (Fig. 1b).

### Manufacture and control of Spikebot

**FIG. 2.**
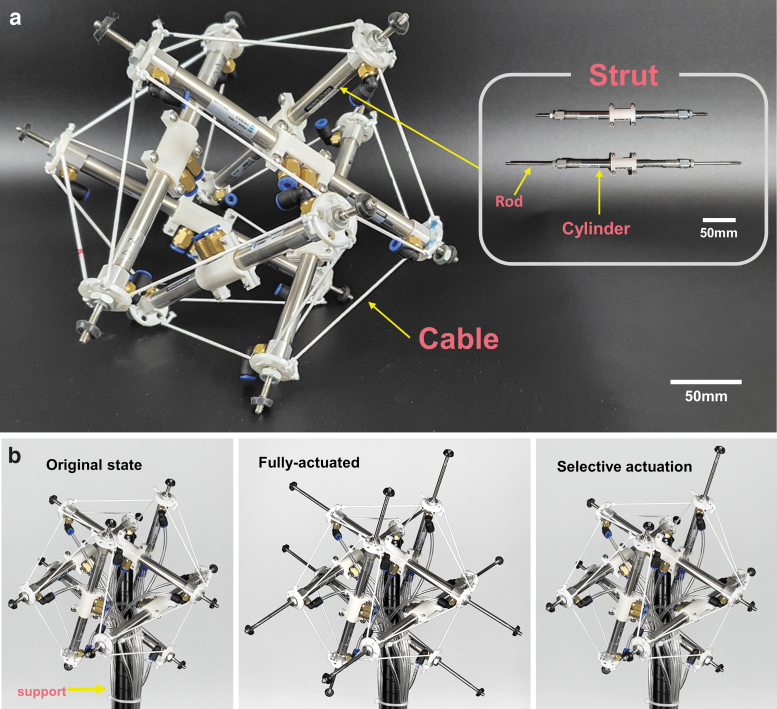
The overall structure of Spikebot and the selective actuation of its struts. **(a)** Spikebot consists of 24 elastic cables and 6 rigid struts with two independent linear pneumatic actuators that can each extend a rod to enable motion. **(b)** Representative actuation states of Spikebot, including the fully retracted (*left*) and fully extended states (*middle*), along with selectively extending a single actuator (*right*).

Figure 2 showcases the overview of Spikebot, which employs a Jessen's icosahedron-shaped tensegrity structure that consists of 6 struts and 24 cables. To manufacture Spikebot, pneumatic linear cylinder actuators with 45 mm stroke (MC10 by SYM, Korea) are purchased and connected to solenoid valves (KS320s by KCC Ltd., Korea) via pneumatic tubes of 2 mm in diameter. A strut is prepared by connecting two of the pneumatic cylindrical actuators with a polylactic acid (PLA) coupler, and a PLA cable holder secures each cable with the same angle and length (Fig. 2a). The struts are connected by elastic cables to uphold the three-dimensional structure, and therefore, the linear extension of the struts results in the spatial expansion of the robot.

A 16-channel relay module (SZH-RLBG-052, by SMG-A, China) is used to control the total of 12 linear actuators within Spikebot (Supplementary Fig. S1), and a compressor (OFS600-8, by Compworld, Korea) is used to generate and control the pneumatic pressure to operate each actuator. In addition, a microprocessor (Arduino Uno) is used to control the relay module and the actuators by serial communication. This control circuit allows for selective actuation of the struts for both their extension and retraction motion and speed (Fig. 2b; Supplementary Fig. S2).

### Geometrical characteristics of Spikebot

**FIG. 3.**
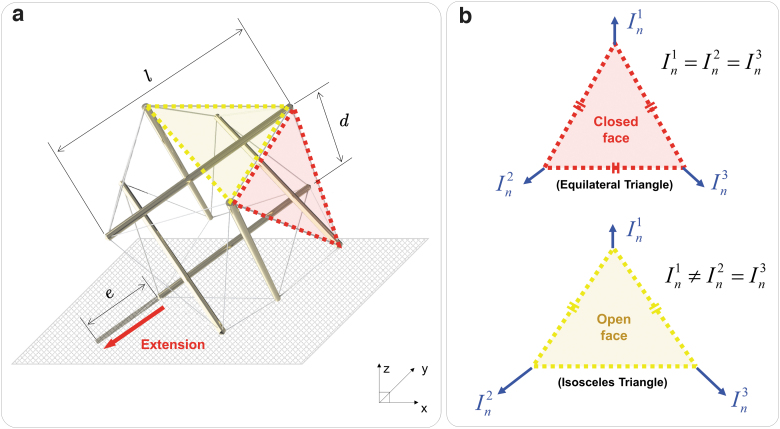
Geometrical characteristics of Spikebot and two distinct types of the face that can serve as the base plane. **(a)** Spikebot is constituted of 20 faces that are shaped as either closed faces (cables connected at all corners) or open faces (cables connected at two corners). The dimension of each face type is determined by the length of the struts *l* and the distance between two parallel struts *d*. A strut can be extended by the length *e*, at the node of the base plane (the face in contact with the ground), to create the thrust necessary for the locomotion. **(b)** Two different base plane types (closed face vs. open face) and the comparison of thrusts in a direction normal to the ground *I_n_*. When the open face serves as the base plane, the ground-normal thrusts *I_n_* varies depending on the actuation node ($I_{n}^{1}\neq I_{n}^{2}=I_{n}^{3}$), that is different from the case of closed face ($I_{n}^{1}=I_{n}^{2}=I_{n}^{3}$).

Spikebot has 20 faces, each characterized either as a closed face (cables connected at all corners) or as an open face (cables connected at two corners), as shown in Figure 3. As Spikebot locomotes, the base plane (the plane in contact with the ground) shifts between each of these 20 faces.

The geometrical characteristics of Spikebot can be quantified from the length of the struts *l*, the distance between two parallel struts *d*, and the extension length of the strut *e* (Fig. 3a). The strut is usually extended from a node of the base plane to create the thrust necessary to roll Spikebot in the opposite direction of the extension.

Depending on whether the base plane is a closed face or an open face, the robot's thrust may induce different types of motion. In detail, as shown in Figure 3b, the closed face is shaped as an equilateral triangle and the thrusts normal to the ground *I_n_* are identical from all three nodes. On the other hand, the open face is nominally an isosceles triangle, giving rise to two distinct amplitudes of the ground-normal thrust *I_n_* from its nodes (Supplementary Fig. S3).

Such unique geometry of base planes determines the motion primitive as well as the controllability of Spikebot, thereby allowing the control strategy of Spikebot to be adapted on the fly.

## Results and Discussion

**FIG. 4.**
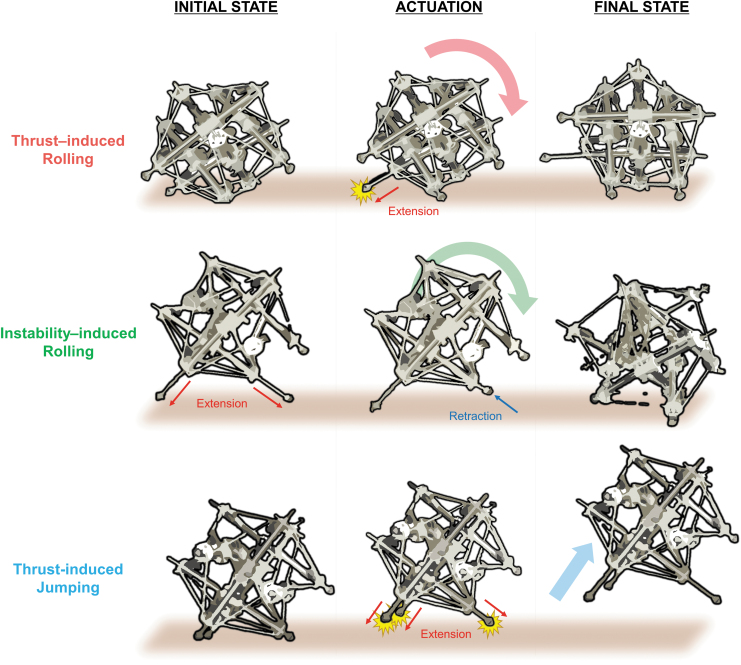
Multi-mode locomotion that Spikebot can execute. Spikebot is capable of three types of locomotion: thrust-induced rolling (*top*), instability-induced rolling (*middle*), and thrust-induced jumping (*bottom*). Thrust-induced rolling is achieved by an extending strut pushing it along a desired direction. Instability-induced rolling is realized as one or two struts retract from the fully extended initial state, allowing the robot to passively tip toward the retracted actuator. Finally, thrust-induced jumping is a result of rapidly extending several actuators, propelling the robot off the ground in a desired direction.

Spikebot can engage in diverse types of locomotion by modulating the sequence and/or speed of strut extensions. In this study, we showcase and mathematically model three different types of locomotion: thrust-induced rolling, instability-induced rolling, and thrust-induced jumping (Fig. 4). Spikebot readily combines these locomotion modes, demonstrating versatility as a mobile robot in several environments.

### Thrust-induced rolling

**FIG. 5.**
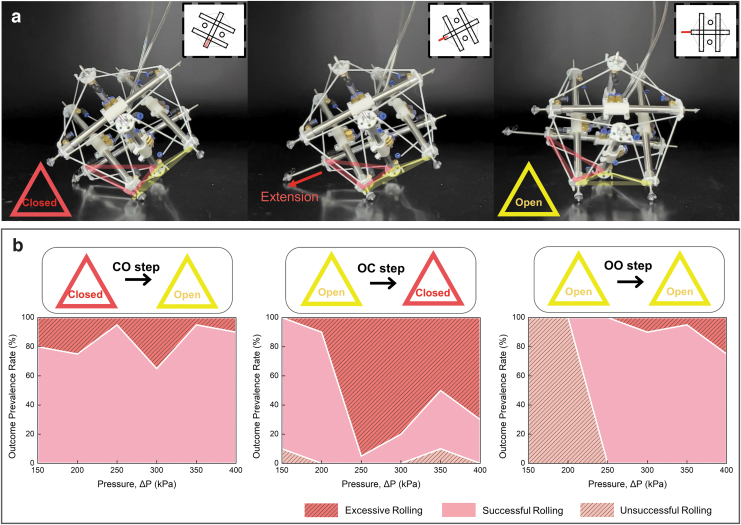
Characteristics of the thrust-induced rolling of Spikebot. **(a)** Timelapse image of Spikebot executing a rolling locomotion by extending a strut. The rolling is referred to as “CO step” since the robot starts on a closed face (depicted as a *red triangle*) and rolls to an open face (depicted as a *yellow triangle*). **(b)** The three canonical rolling motions (CO step, OC step, and OO step) and their success rate as a function of actuator pressure. The actuator pressure governs the speed of extending struts, and the resulting success rate is highly dependent on the types of rolling motion.

Actuation of a strut from the base plane creates thrust sufficient to roll Spikebot about its center of gravity (Fig. 5). This thrust-induced rolling motion is different from widespread rolling mechanisms of tensegrity robots that harness structural instabilities. Namely, since Spikebot's thrust-induced rolling is governed by the force induced from extending struts to impact the ground, Spikebot maintains its structural stability and can therefore fully utilize its internal space for payloads. Such structural stability during locomotion also benefits its ability to withstand external collision by elastic tension cables absorbing the impact energy.

Moreover, since the speed and stroke of each strut can be selectively controlled, Spikebot diversifies and unlocks the rolling locomotion of tensegrity robots. In detail, Spikebot can execute rolling motion regardless of its base plane types (closed face vs. open face). In general, icosahedron-shaped tensegrity robots have been known for three rolling types depending on their initial base plane and final base plane after a rolling motion: closed base-to-open base (CO step), open base-to-closed base (OC step), or open base-to-open base (OO step).

Among these three steps, CO steps are particularly difficult for icosahedron tensegrity robots to accomplish by structural deformation because the closed-base planes are highly stable attributed to their equilateral triangle shape. However, as shown in Figure 5a, Spikebot is capable of realizing all three steps, including the CO step, because the extending strut creates sufficient thrust to overcome the structural stability granted from the closed base plane.

Pressure applied to extending strut is an important control factor to achieve reliable rolling motion. Figure 5b depicts the outcome prevalence rate of the thrust-induced rolling with respect to the pressure applied to the extending strut that is directly related to the extension speed (Supplementary Fig. S2). Each rotation step is accomplished by extending a single strut located opposite side in the direction of rolling, and the successful rolling case is defined as when the base plane of Spikebot is switched to the adjunct face. Similarly, the excessive rolling case and the unsuccessful rolling case are defined when the base plane is switched more than once and when the base plane is unchanged, respectively.

The experimental results reveal that the rolling efficiency of Spikebot varies with the rolling types, and CO step is the most reliable among the total three rolling types with the maximum outcome prevalence rate of >95%. The effect of the inlet pressure on achieving the CO step is less notable than OC step and OO step.

On the other hand, both OC step and OO step are highly dependent on the inlet pressure, with each type of rolling still having a range of optimal inlet pressures that can achieve outcome prevalence rate >90%. So, by modulating the inlet pressure according to the initial base plane and the rolling direction, the thrust-induced rolling of Spikebot can be subsequently executed to realize continuous rolling motion.

### Instability-induced rolling

**FIG. 6.**
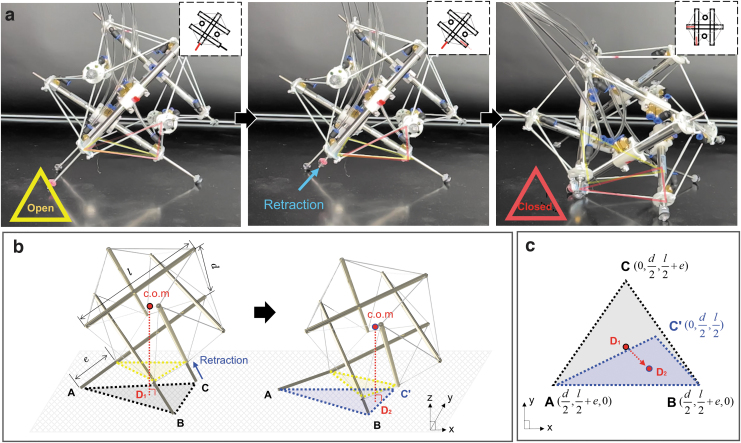
Overview of the instability-induced rolling of Spikebot. **(a)** Timelapse image of Spikebot rolling from the base plane of fully extended struts. Retracting a previously extended strut gives rise to the rolling motion from an open face (emphasized by a *yellow triangle* superimposed on the image) to a closed face (emphasized with a *red triangle*). **(b)** Schematic of the locomotion principle that entails the augmentation of the base plane and shift of the center of mass (from *D*_1_ to *D*_2_) with respect to the base plane. **(c)** Illustration of the base plane that shows the shift of c.o.m in response to the strut retraction. The c.o.m shift ($\bar{D_{1}D_{2}}$) becomes apparent with increasing extension length *e* and decreasing strut distance *d* (Supplementary Fig. 4), thereby creating pronounced rolling motion.

Spikebot can additionally realize rolling motion by harnessing geometrical instability (Fig. 6). The instability can be induced from the initial fully actuated state of Spikebot—where all struts on the base plane are extended outward—by retracting one strut (Fig. 6a). As a result of the strut retraction, Spikebot rotates about the remaining two extended struts and eventually impacts the ground with sufficient momentum to roll to the adjacent face.

Figure 6b depicts the change of the base plane (from ΔABC to ΔABC′) and the resultant shift of the center of mass (c.o.m) within the base plane (from *D*_1_ to *D*_2_). Because Spikebot is skewed in contact with the re-established base plane ΔABC′, the location of its c.o.m within the base plane *D*_2_ is notably shifted toward the edge, thereby spurring the subsequent rolling motion.

Compared with thrust-induced rolling, instability-induced rolling is advantageous because the motion is independent of the actuation force (i.e., pressure). In detail, the instability is gained from reshaping the orientation and area of the base plane, demanding no actuation force afterward, ultimately resulting in an energy-efficient rolling motion. However, the instability-rolling of Spikebot is limited in its versatility because the rolling motion is only achieved when the base plane is on the open face.

The success rate of the instability-induced rolling marks 100% with the open face as the base plane, but on the other hand, Spikebot is lack of creating enough momentum to roll when the closed face serves as the base plane. Figure 6c illustrates the shift of c.o.m, in response to the instability, where the shifting length $\bar{D_{1}D_{2}}$ directly scales with the momentum granted to Spikebot after the initial rotational motion.

Since the shifting length $\bar{D_{1}D_{2}}$ is a function of the distance between two parallel struts *d* and the extension length of the strut *e* (Supplementary Fig. S4), we note that more pronounced rolling can be achieved by increasing the ratio e∕d.

### Thrust-induced jumping

**FIG. 7.**
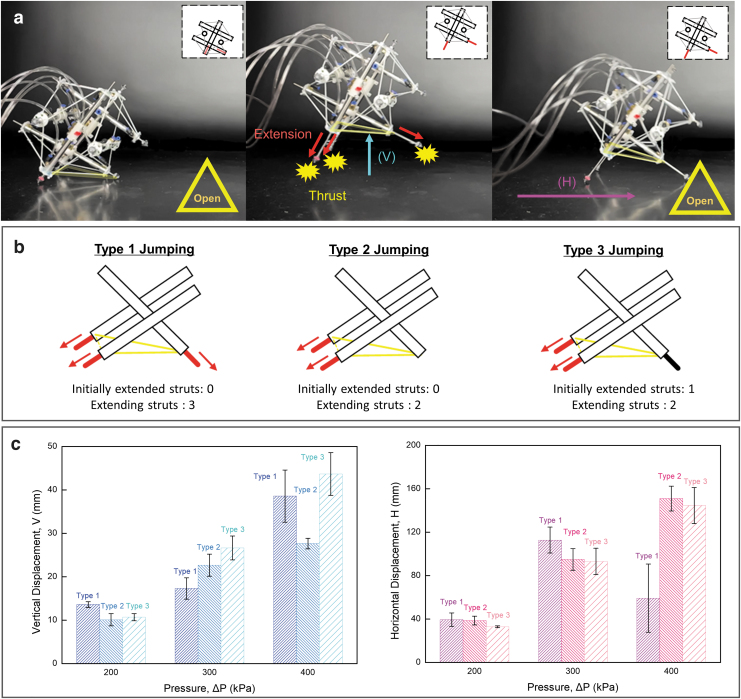
Characteristics of the thrust-induced jumping of Spikebot. **(a)** Timelapse image of Spikebot that jumps in vertical (V) and horizontal directions (H). The base plane is retained as an open face. **(b)** Illustration of three different types of jumping motion, initiated from different strut configurations. **(c)** Travel distance in vertical and horizontal directions obtained from each jumping locomotion as a function of actuator pressure. Increased pressure leads to larger displacements, although the benefit depended strongly on the jumping type. The *error bars* indicate the standard deviation of six trials.

When two or more actuators are extended from the base plane, the resultant thrust is sufficient to cause Spikebot to jump, as shown in Figure 7. Thrust-induced jumping produces horizontal *H* and vertical *V* displacement components, yet the base plane remains unchanged (Fig. 7a). In this study, Spikebot's jumping is examined only when an open face is serving as the base plane.

In the case of jumping with a closed face as the base plane, the *V* component is negligible compared with that when an open face serves as the base plane. Such difference in jumping ability is presumably due to a lower ground-normal thrust *I_n_* created from the base plane when a closed face is in contact with the ground (Supplementary Fig. S4).

With an open face serving as the base plane, the jumping motion of Spikebot can be achieved with various strut configurations. The strut configurations are determined by whether each strut is retracted or extended at the initial state before jumping, and we examine three types of strut configurations in this study (Fig. 7b).

Type 1 jumping is executed by simultaneously extending all struts on the ground, and Type 2 jumping is controlled by extending only two struts while the other strut remains retracted. Type 3 jumping is similar to Type 2 jumping, but the previously retracted strut remains extended. Such variations of strut configuration are designed to control the jumping characteristics of Spikebot with a focus on its horizontal *H* and vertical *V* displacement.

Figure 7c summarizes jumping characteristics of Spikebot with the actuation schemes cited earlier. Both *V* and *H* components are measured for five independent jumps with increasing inlet pressure that directly scales with the thrust for jumping. In general, the higher the thrust, the greater the value of both components.

However, the Type 1 jumping scheme reveals that the *H* component can be reduced with increasing thrust, presumably because of the rod in the vertex that creates thrust in the opposite direction. In detail, above a certain pressure threshold, the front extending strut in the locomotion direction pushes off the ground, inducing a moment that raises the front of SpikeBot.

As a result, the thrust produced from the rear extending struts primarily contains a vertical component, rather than a horizontal component, thereby reducing *H* and increasing *V*. Such hindrance of *H* translation is mitigated in the Types 2 and 3 jumping schemes. Likewise, Spikebot's ability to translate in both *V* and *H* direction expands the locomotion space of the tensegrity robot to three dimensions, enhancing the capability of Spikebot as a mobile robot.

### Demonstrations

**FIG. 8.**
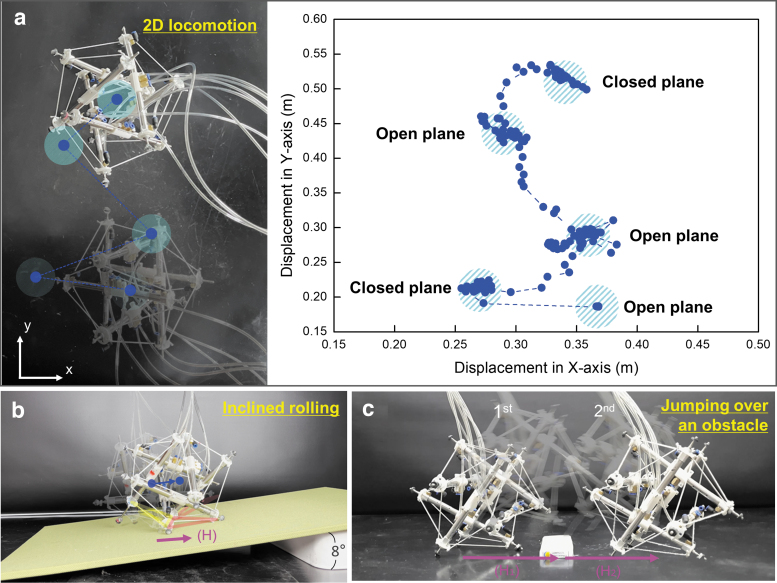
Demonstration of Spikebot locomoting various environments. **(a)** Spikebot maneuvering over flat ground in both *x*- and *y*-directions. *Left*: *top-down* view of the experiment. *Right*: The path traced by Spikebot's center of gravity during the locomotion sequence, along with annotations emphasizing which face was in contact with the ground during periods of relative equilibrium. **(b)** Inclined locomotion of Spikebot achieved from the thrust-induced rolling of “OC step.” Such incline motion can be also realized by thrust-induced jumping. **(c)** Spikebot that overcomes an obstacle through a couple of thrust-induced jumping motions. Combinations of in-plane locomotion and jumping are expected to unlock the locomotion space of Spikebot, spurring its utility as a mobile robot.

Spikebot is adaptable to various environments because it can modulate between diverse locomotion modes, maintaining mobility in various situations. We showcase the consistent mobility of Spikebot as it overcomes several obstacles in controlled settings (Fig. 8; Supplementary Movie S1). Figure 8a reveals that Spikebot rolls in two-dimensional space by repetition of thrust-induced rolling.

Each rolling motion is rapid and achieved with 100% success rate, demonstrating Spikebot's better ability to explore a large area of exploration than the conventional tensegrity robot (Supplementary Fig. S5). Moreover, since the excessive rolling mechanism can increase the locomotion speed while the instability-induced rolling can serve as another locomotion principle, Spikebot is expected to enhance the efficiency of two-dimensional locomotion by the combination of diverse thrust- and instability-induced rolling motions.

We also demonstrate Spikebot's ability to traverse an inclined slope, which has been a challenging task for several deformation-driven tensegrity robots. Figure 8b shows the inclined rolling of Spikebot against a slope of 8° by utilizing thrust-induced rolling.

Such inclined locomotion is difficult to realize by instability-induced rolling because the inclined base plane reduces the shift of c.o.m. during actuation, but thrust-induced jumping shows comparable performance with thrust-induced incline rolling. Increasing the extension speed of struts is expected to assist Spikebot in locomoting higher angle slopes with the enhanced impact energy.

Lastly, we examine the effectiveness of three-dimensional space locomotion of Spikebot. Figure 8c shows that Spikebot can leap over an obstacle along its locomotion path via thrust-induced jumping. As the locomotion principle of thrust-induced rolling suggested (Fig. 7c), the jumping motion simultaneously creates both vertical and horizontal locomotion and allows Spikebot to leap over an object.

Such locomotion in three-dimensional space is expected to upscale the practical utility of Spikebot, which can overcome irregular shapes and obstacles of terrain. In sum, robotic demonstrations herein suggest that Spikebot can be a viable robot to explore diverse terrestrial environments because of its diverse locomotion modes that complement current tensegrity robots.

## Conclusions

Inspired by Tetraodontidae, we have herein presented Spikebot, a tensegrity robot that is capable of selectively actuating each strut to spatially expand, locomoting in three-dimensional space by rolling and jumping. Three different motion primitives are investigated, and their combination allows increased options for path finding and subsequent realization of efficient and dynamic navigation over varied terrain.

Such path-finding ability is expected to unlock the functionality of the robot in exploring unmanned space and severe environments. One of the noteworthy benefits of Spikebot is its ability to maintain an unaltered internal space, by expanding solely on the external side. This unique characteristic creates a stable and protected internal payload volume during operation, for carrying scientific payloads or navigation sensors, for example.

We demonstrate SpikeBot's ability to explore multi-dimensional space in several controlled settings with varied terrain. In future work, we plan to increase Spikebot's utility as a field robot by implementing an untethered actuation system. We surmise the realization of an untethered actuation system could be accomplished by replacing pneumatic linear actuators with battery-powered actuators paired with a bistable mechanism capable of rapidly releasing stored elastic energy.

In addition, field compatibility should be tested by deploying the robot with sample payloads, under several dynamic deployment strategies (e.g., mid-air discharge). In all cases, we contend that the design and motion primitives of Spikebot covered herein will facilitate the practical use of tensegrity robots on the field.

## Supplementary Material

Supplemental data

Supplemental data
