# Supplementary material for: Spikebot: A Multigait Tensegrity Robot with Linearly Extending Struts
Source: Soft Robot. 2024 Apr 8;11(2):207–17. doi: 10.1089/soro.2023.0030 (PMC11035858; doi:10.1089/soro.2023.0030)
Supplement: Supplemental data [file Suppl_Data.docx]

**Spikebot: a Multigait Tensegrity Robot of Linearly Extending Struts**

Jinwook Jeong^a^, Injoong Kim^a^, Yunyeong Choi^a^, Seonghyeon Lim^a^, Seungkyu Kim^a^, Hyeongwoo Kang^a^, Dylan Shah^b^, Robert Baines^b^, Joran W. Booth^b^, Rebecca Kramer-Bottiglio^b^, and Sang Yup Kim^a*^

^a^Department of Mechanical Engineering, Sogang University, Seoul, Republic of Korea

^b^School of Engineering & Applied Science, Yale University, New Haven, CT 06511, USA

*Corresponding author. Department of Mechanical Engineering, Sogang University, Seoul 04107, Republic of Korea

Email address: [sangyupkim@sogang.ac.kr](mailto:sangyupkim@sogang.ac.kr) (S. Y. Kim)

**Contents**

Supplementary Figure 1

Supplementary Figure 2

Supplementary Figure 3

Supplementary Figure 4

Supplementary Figure 5

Supplementary Movie 1 (separate file)


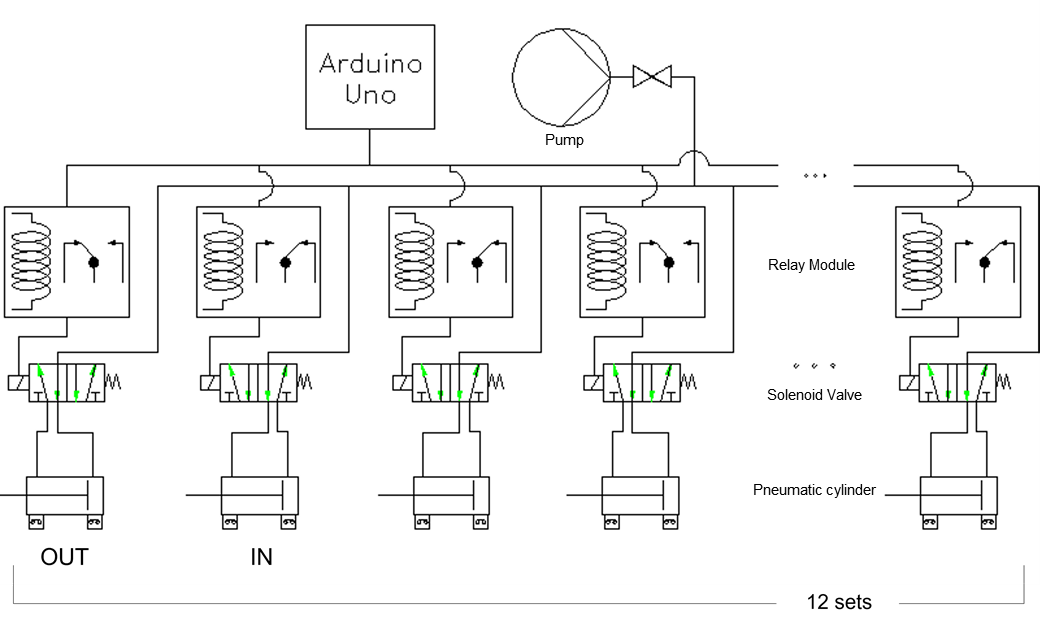


**Supplementary Figure 1. Diagram of the control circuit of Spikebot.** A total of 12 pneumatic actuators are individually controlled via 12 solenoid valves connected to a 16-channel relay module.

**Supplementary Figure 2. The extension speed of a strut as a function of the inlet pressure.** Increasing inlet pressure linearly scales the actuation speed of the strut. The actuation speed is calculated as an average speed from the initial nominal length 240 mm($l$) to 285 mm($l+e$)


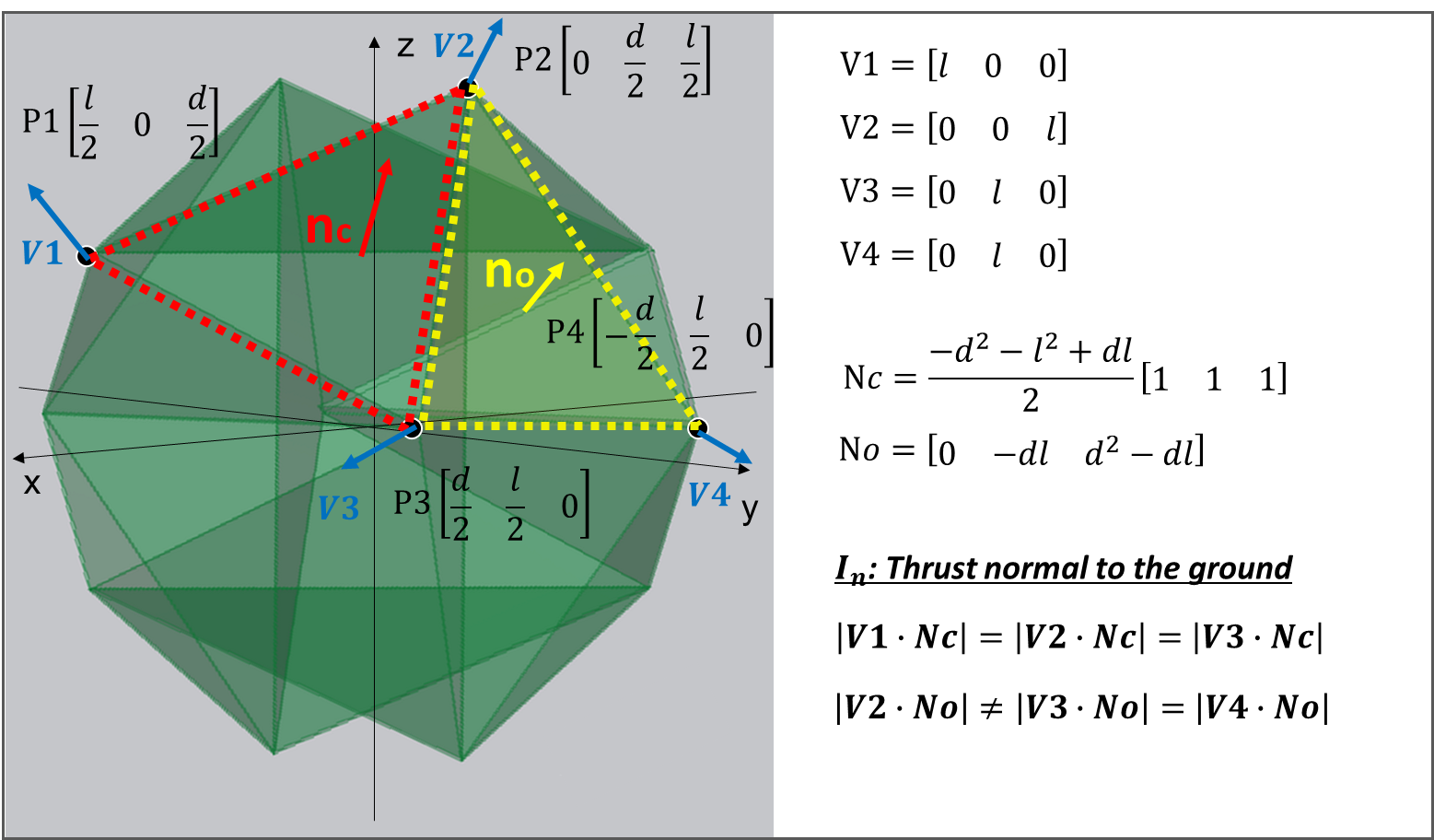


**Supplementary Figure 3. Geometrical characteristics of the closed and open faces of Spikebot.** The difference in the face shapes results in different levels and angles of thrusts when a strut is extended. In detail, the closed face is shaped as an equilateral triangle, and therefore, the thrust normal to the ground is identical for all three nodes whereas the ground-normal thrust of open face varies for each node, since it has an isosceles triangle shape.

**
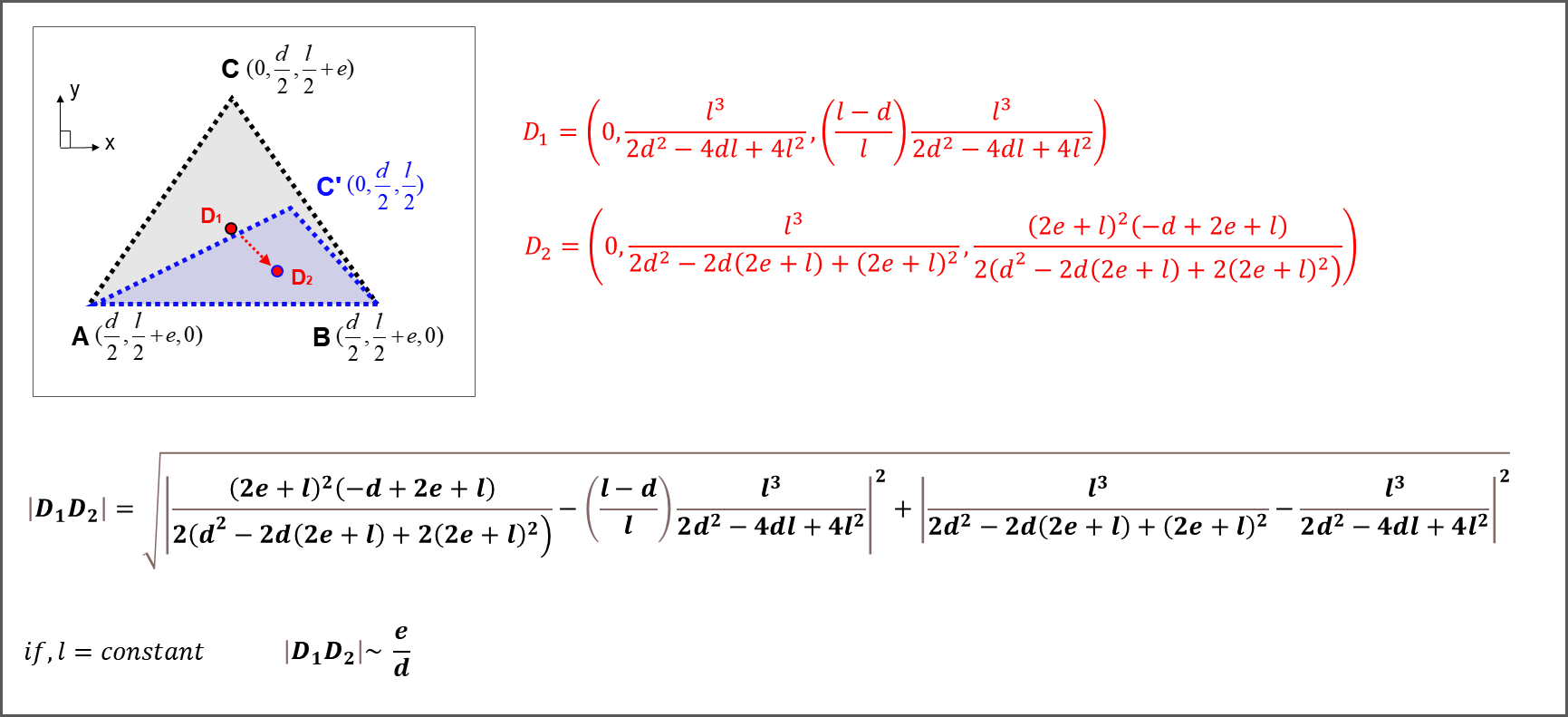
**

**Supplementary Figure 4. Calculation of how the center of mass shifts with respect to the base plane, upon the retraction of a strut for instability-induced rolling.** The position vectors of $D_{1}$ and $D_{2}$ are the center of mass projected onto the base plane $\triangle ABC$ and $\triangle ABC'$, respectively. The shifting distance $\overline{D_{1}D_{2}}$ is determined by the length of the extension $e$ and the distance between two parallel struts $d$.


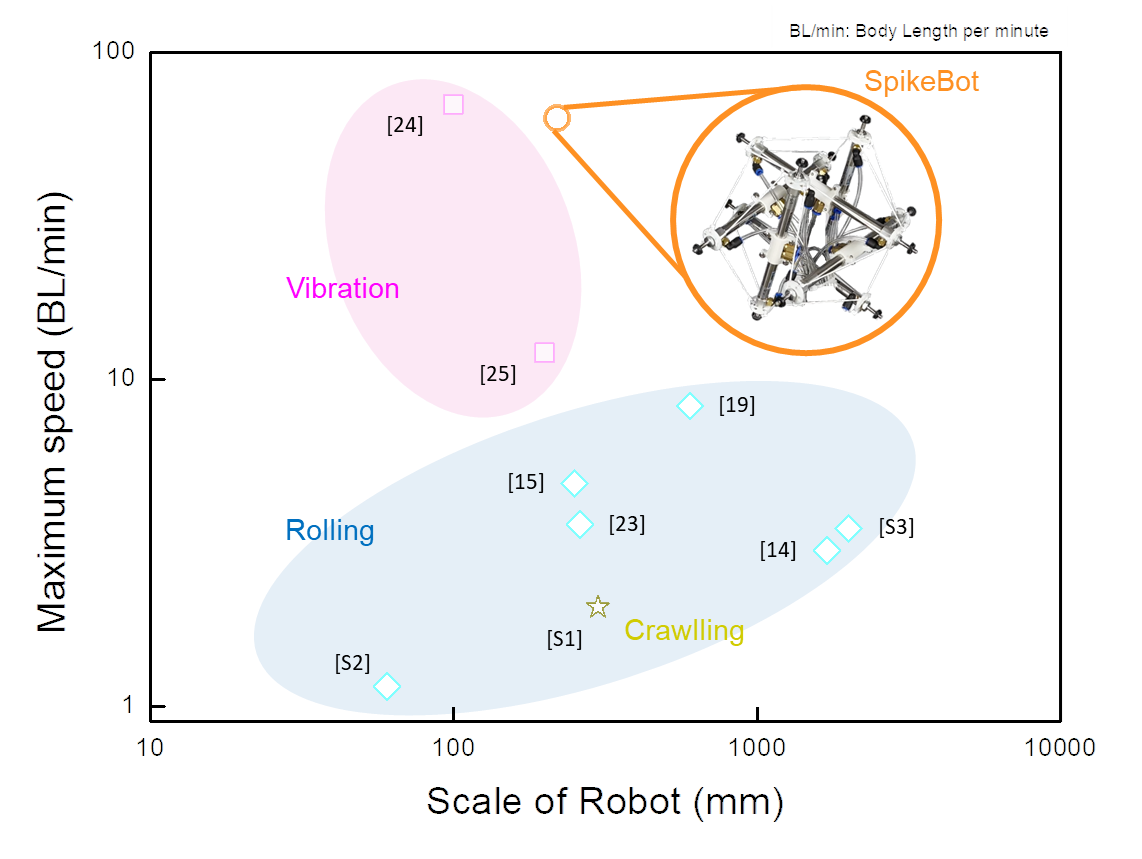


**Supplementary Figure 5. The maximum speed of tensegrity robots as a function of their scale.** The robot’s speed unit, BL/min, is calculated by dividing the speed of the robot by the length of the strut. Rolling-driven tensegrity robots show relatively slower locomotion than vibration-driven tensegrity robots. SpikeBot showcases its rapid locomotion in addition to the diverse locomotion modes.

**Reference**

**S**1. Paul C, Valero-Cuevas FJ, Lipson H. Design and control of tensegrity robots for locomotion. IEEE Transactions on Robotics 2006;22(5):944–957; doi: 10.1109/TRO.2006.878980.

S2. Wang Z, Li K, He Q, et al. A Light-Powered Ultralight Tensegrity Robot with High Deformability and Load Capacity. Advanced Materials 2019;31(7):1806849; doi: 10.1002/ADMA.201806849.

S3. Vespignani M, Friesen JM, Sunspiral V, et al. Design of SUPERball v2, a Compliant Tensegrity Robot for Absorbing Large Impacts. IEEE International Conference on Intelligent Robots and Systems 2018;2865–2871; doi: 10.1109/IROS.2018.8594374.
